# Stereotypical patterns of epileptiform calcium signal in hippocampal CA1, CA3, dentate gyrus and entorhinal cortex in freely moving mice

**DOI:** 10.1038/s41598-019-41241-x

**Published:** 2019-03-14

**Authors:** Xin Zhang, Zhihong Qiao, Nannan Liu, Lili Gao, Liangpeng Wei, Aili Liu, Zengguang Ma, Feifei Wang, Shaowei Hou, Jisheng Li, Hui Shen

**Affiliations:** 1 Laboratory of Neurobiology, School of Biomedical Engineering, Tianjin Medical University, Tianjin, China; 2 VCANBIO biological Resources Storage (Tianjin) Co. Ltd, Tianjin, China

## Abstract

Epilepsy is a multi-etiological brain dysfunction syndrome. Hippocampal neuronal damage induced by seizures may be one of the causes leading to cognitive impairment, but the underlying mechanism remains to be further elucidated. The kainic acid (KA) model of temporal lobe epilepsy is widely used in understanding of the epileptogenesis. Fiber photometry is a signal detection technology suitable for recording calcium activity of neurons in the deep brain of freely moving animal. Here, we used the optical fiber-based method to monitor the real-time neuronal population activities of freely moving mice after subcutaneous injection of KA. We observed that KA administration led to one to three kinds of stereotypical patterns of epileptiform calcium activity in CA1, CA3, and dentate gyrus (DG) of the hippocampus, as well as the entorhinal cortex (EC). There were three kinds of waves in the hippocampal CA1, which we named wave 1, wave 2 and slow flash. Wave 1 and wave 2 appeared in both the CA3 and DG regions, but the EC only showed wave 1. In these epileptiform calcium signals, we observed a high amplitude and long duration calcium wave as a part of wave 2, which resembled cortical spreading depression (CSD) and always appeared at or after the end of seizure. Because the same characteristic of epileptiform calcium signal appeared in different brain regions, calcium signal may not exist with region specificity, but may exhibit a cell type specific manner. Thus, our work provides a support for the pathogenesis of epilepsy and epileptiform signal transmission research.

## Introduction

Epilepsy is a multi-etiological brain dysfunction syndrome, affecting almost 70 million people around the world^1^. Seizures have caused severe physical and mental torture to patients themselves, and some patients even face risks such as cognitive function and affective disorders^2^. Hippocampal neuronal damage induced by seizures may be one of the causes leading to cognitive impairment, but the underlying mechanism remains to be further elucidated. It is generally believed that epilepsy is related to abnormal excitement and desynchronization of neurons^3^. The calcium hypothesis of epilepsy^4^ points out that when the intracellular calcium concentration is beyond the normal level, yet dose not reach the extent of calcium overload to generate excitotoxicity, the abnormal increase of calcium concentration may cause transient or persistent changes in neural plasticity. These neuronal pathological changes, in the long run, may results in abnormal discharge of neurons which ultimately causes epilepsy. In recent years, there has been an increasing interest in calcium activities^5,6^ in the brain of freely behaving mice, for instance, in neurological disease models including depression^7^, autism^8^ and epilepsy^9^. Previous research^10^ had shown the pathological calcium activity in hippocampal CA1 preceding convulsive motor seizures. In addition, Vikaas *et al*. observed that seizures induced by optogenetically stimulation produced large calcium signals in all studied cell types in primary motor cortex^9^. However, although there were some researches did record epileptiform calcium signal in freely moving animals, few work has recorded the signals in different regions in the same research, and there is also specially lack of analysis and classification of the stereotypical patterns of calcium signals in different brain areas.

KA is a direct agonist of glutamatergic KA receptors. The binding of KA to its cognate receptor elicits robust depolarization of cells, one of the main phenomena of temporal lobe epilepsy^11,12^. Seizure events induced by KA injection is a widely accepted model of human temporal lobe epilepsy and treatment-resistant epilepsy^13–15^.

**Figure 1:**
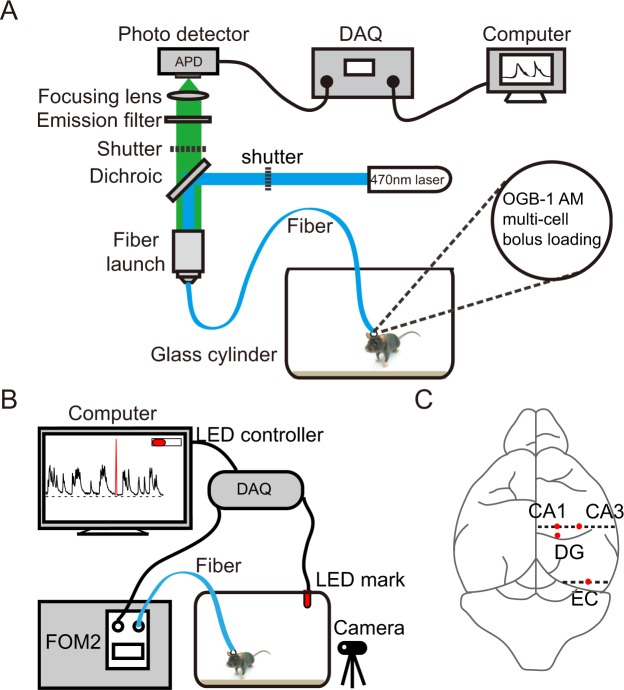
Fiber photometry setup. (**A**) Schematic diagram of calcium signal recording system. The wavelength of emission filter and the dichroic filter was 472 nm ± 30 nm and 520 nm ± 36 nm. (**B**) Calcium signal and behavior of freely moving mice were recorded respectively and simultaneously, synchronized them with a LED mark to off-line analyze the correlation. (**C**) Schematic diagram showing the site on brain surface of optical fiber implanted in hippocampal CA1, CA3, DG and EC stained with OGB-1 AM.

In this study, we combined well established assessments of seizure activity such as Racine rules^16^ with functional calcium signals recording of calcium indicator Oregon green 488 BAPTA-1 AM (OGB-1 AM) in the hippocampal CA1, CA3, DG and EC in freely behaving mice subcutaneously administrated of KA. To analyze the relationship between epileptic seizures and brain calcium activity, we recorded calcium signals and behavioral videos simultaneously and synchronized them with a LED mark (Fig. 1). Calcium recording based on fiber photometry has the advantage of recording in deep brain regions of freely behaving mice, with small physical damage to brain tissue and simple experimental operation^17,18^.

## Results

### Epileptiform Calcium Waves in Freely Moving Mice after KA Administration

KA administration induces seizures by direct activation of KA receptors, causing abnormal discharge of neuronal cells in the limbic structures^11^. Previous experiments had shown that single KA injection of 15 mg/kg can induce convulsive motor seizure in C57BL/6 mice.

**Figure 2:**
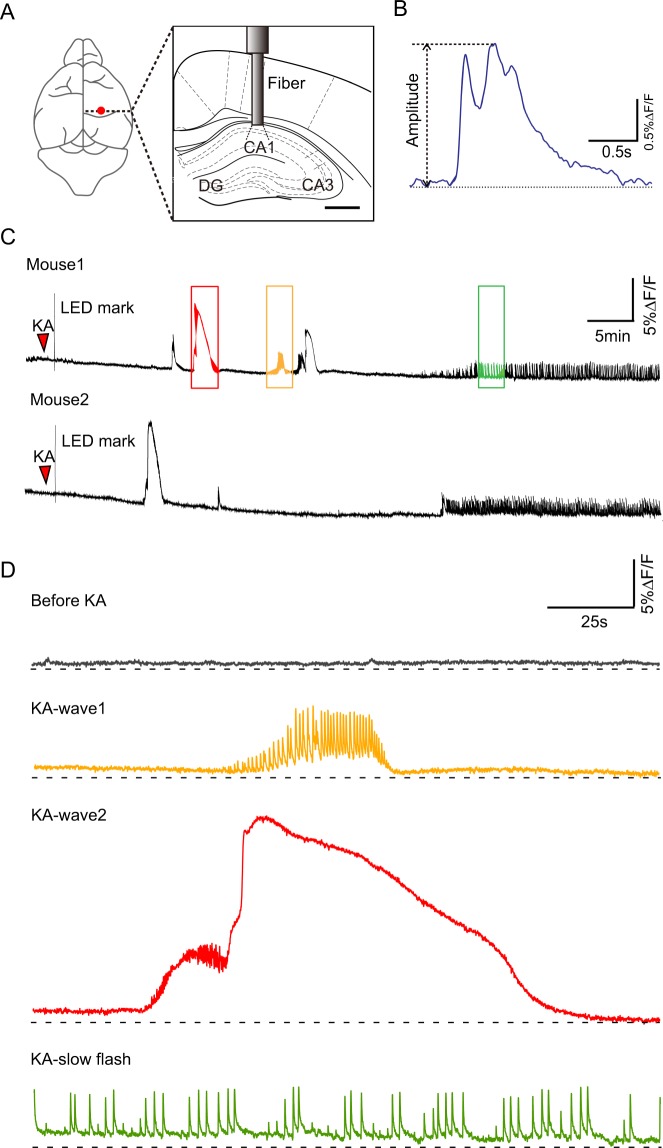
Specific calcium signal in hippocampal CA1. (**A**) Schematic diagram showing the optical fiber implanted stereotaxical center in hippocampal CA1, CA3 and DG. (**B**) Amplitude analyze for a calcium signal. (**C**) Examples showing the epileptiform calcium signal of CA1 in freely behaving mice lasted 1 h. There were three different types, that as wave1 (Orange box), wave2 (Red box) and slow flash (Green box). (**D**) Examples showing intercepting and amplifying epileptiform calcium signal before (Gray) and after KA administration, wave1 (Orange), wave2 (Red) and slow flash (Green).

We stained with the calcium indicator OGB-1AM in hippocampal CA1, CA3, DG or EC and applied the optical fiber-based approach to record population calcium activity. To investigate the correlation between the population calcium activities and the behaviors, we recorded signals of freely behaving mice in a transparent round glass cylinder (Fig. 1B). Behaviors were recorded with a camera which was placed in front of the cylinder. The recordings were started at least 1.5 h after anesthesia was ended (Fig. 2C).

We recorded four different waveforms in hippocampal CA1 and we named them before KA (Fig. 2D, Gray), wave1 (Fig. 2D, Orange), wave2 (Fig. 2D, Red) and slow flash (Fig. 2D, Green). The latter three were epilepsy related.

Before KA administration, calcium activities were observed as irregular pulse-like or continuous calcium signals with small amplitude (Fig. 2D, gray, before KA). The irregular pulse-like signals had fewer occurrences in the recording process and the duration of a single calcium transient was about 1.5 s.

We observed that most epileptiform calcium activities were associated with behavioral seizures. By referring to Racine scale (see Methods, Seizure assessment), we classified the seizures of mice into six stages, from no changes in behavior (stage 0) to generalized tonic-clonic seizures with loss of posture and falling (stage 5). A seizure stage 3 or higher was classified as a convulsive motor seizure (CMS). However, because of the individual differences of mice, different mice might have different levels of seizures by injecting the same dose of KA. When the same calcium waves appeared at different times in the same mouse or in different mice, the corresponding seizures would change in a certain range of levels.

After KA administration, wave1 and wave2 mainly appeared in the first 40 min. Wave1 was observed as a small calcium wave with high frequency oscillation and the duration was about 50 s. When the wave1 appeared, the mice represented as sudden behavioral arrest and motion less staring (stage 1) to intermittent ear twitches or hiccups (stage 2). Wave2 was observed as calcium build-up followed by large calcium wave and the duration was about 125 s. During calcium build-up, the mice represented as intermittent ear twitches or hiccups (stage 2), to generalized clonic seizures with rearing (stage 4), reaching CMS. Calcium signals developed to constant slow flash pattern at ~40 min after KA administration. Slow flash was cluster low frequency pulse and the duration of single calcium transient was about 1.5 s. The mice represented persistent seizure, that as clonus of the head or forelimbs and tail rigidity (stage 3). There may be two kinds of waves coming together.

Both the hippocampus and EC are very important in epilepsy^19^, we also recorded the the calcium signals and corresponding behaviors of hippocampal CA3, DG and EC before and after KA administration.

### Stereotypical Patterns of Epileptiform Calcium Activities in hippocampal CA1, CA3 and DG and EC of Freely Moving Mice after KA Administration

**Figure 3:**
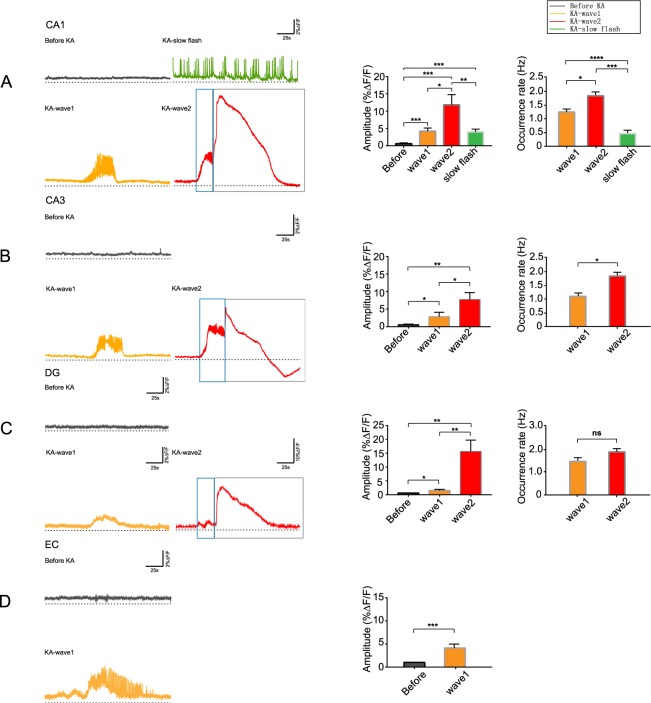
Specific calcium signal in hippocampus and EC. (**A**) Four kinds of calcium activities in hippocampal CA1. The amplitudes of these four kinds of calcium signals were as follows. Before KA: 0.65 ± 0.04% ∆F/F, wave1: 4.25 ± 0.35% ∆F/F, wave2: 11.83 ± 1.21% ∆F/F, slow flash: 4.03 ± 0.32% ∆F/F; before vs. wave1, ***p = 0.0001; before vs. wave2, ***p = 0.0002; before vs. slow flash, ***p = 0.0002; wave1 vs. wave2, *p = 0.0031; wave2 and slow flash, **p** = **0.0014; n = 4 mice, Paired t-tests. The occurrence rates were as follows. Wave1: 1.06 ± 0.06 Hz, wave2: 1.66 ± 0.14 Hz, slow flash: 0.28 ± 0.03 Hz; wave1 vs. wave2, *p = 0.0108; wave1 vs. slow flash, ****p  < 0.0001; wave2 vs. slow flash, ***p = 0.0002; n = 4 mice, Paired t-tests. (**B**) Three kinds of calcium activities in hippocampal CA3. The amplitudes of these three kinds of calcium signals were as follows. Before KA: 0.54 ± 0.11% ∆F/F, wave1: 2.86 ± 0.64% ∆F/F, wave2: 7.73 ± 1.45% ∆F/F; before vs. wave1, *p = 0.0148; before vs. wave2, **p = 0.0043; wave1 vs. wave2: *p = 0.02; n = 4 mice, Paired t-tests. The occurrence rates were as follows. Wave1: 0.92 ± 0.07 Hz, wave2: 1.68 ± 0.19 Hz; wave1 vs. wave2, *p = 0.0329; n = 4 mice, Paired t-tests. (**C**) Three kinds of calcium activities in hippocampal DG. The amplitudes of these three kinds of calcium signals were as follows. Before KA: 0.61 ± 0.04% ∆F/F, wave1: 1.53 ± 0.21 ∆F/F, wave2: 15.63 ± 2.04% ∆F/F; before vs. wave1, *p = 0.018; before vs. wave2, **p = 0.006; wave1 vs. wave2: **p = 0.0077; n = 4 mice, Paired t-tests. The occurrence rates were as follows. Wave1: 1.27 ± 0.13 Hz, wave2: 1.68 ± 0.54 Hz; wave1 vs. wave2, *p* = 0.51; n = 4 mice, Paired t-tests. (**D**) Two kinds of calcium activities in EC. The amplitudes of these two kinds of calcium signals were as follows. Before KA: 1.04 ± 0.05% ∆F/F, wave1: 4.15 ± 0.37% ∆F/F; before vs. wave1, ***p = 0.0009; n = 5 mice, Paired t-tests. And the occurrence rate of wave1 was 1.31 ± 0.29 Hz. Wave2 was divided into two parts, namely build-up (blue box) and CSD-like wave (grey box).

As expected, we recorded population calcium activities in CA3, DG and EC stained with the calcium indicator OGB-1AM of freely moving mice (Fig. 3B~D left). The calcium signals in these three regions were similar to those in CA1 before KA administration (Fig. 3, Gray). After KA administration, CA3, DG showed same stereotypical patterns wave1 (Fig. 3, Orange) and wave2 (Fig. 3, Red). In particular, the amplitude of wave2 in CA3 declined below the baseline and gradually recovered which was different from other regions (Fig. 3B, Red). The behaviors of mice (CA3, DG) were similar to those of CA1 when wave1 and wave2 appeared. Specifically, slow flash only appeared in CA1 (Fig. 3A, Green) and there was only wave1 in EC region (Fig. 3D, Orange). When wave1 appeared in EC, mice represented gradually from stage 1 to CMS with time.

On average, the amplitudes of the calcium transients in CA1 (Fig. 3A), CA3 (Fig. 3B), DG (Fig. 3C) and EC (Fig. 3D) after KA administration were significantly higher than those before KA.

Then we analyzed the occurrence rate (the numbers of calcium transients per second) of the three kinds of epileptiform calcium waves. The occurrence rates of the obvious pulse-like oscillation parts were calculated of wave1s. The occurrence rate calculations of wave2s were the parts before the wave rose (Fig. 3, blue box; calcium build-up) which were also high frequency oscillation.

In hippocampal CA1, the occurrence rate of wave1 was similar to wave2 (Fig. 3A). But the occurrence rates of wave1 and wave2 were both higher than that of slow flash (Fig. 3A). In hippocampal CA3, the occurrence rates of wave1 and wave2 were similar but still had certain differences (Fig. 3B). And in DG region, there was little difference between the occurrence rates of wave1 and wave2 (Fig. 3C). However, there was no significant difference in the occurrence rates of same waveforms in different brain regions.

**Figure 4:**
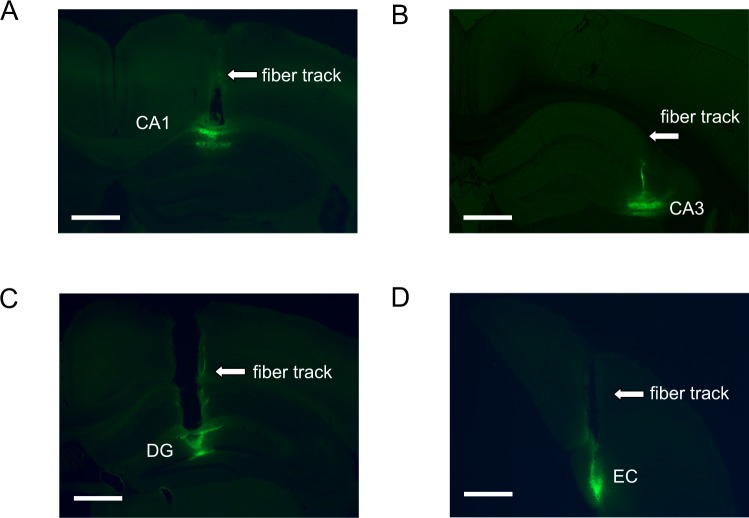
Accurate localization of histology and fluorescence images. Brain samples documented the OGB-1AM staining and confirmed the relative position of the optical fiber in hippocampal CA1 (**A**), CA3 (**B**), DG (**C**) and EC (**D**). The bar is 0.5 mm.

After recording, all experimented mice were perfused transcardially with 4% paraformaldehyde (PFA) in phosphate-bufferedsaline (PBS) to document the OGB-1AM staining and confirm the relative position of the optical fiber (Fig. 4A~D).

### Cortical Spreading Depression-like Events

We recorded large and prolonged (~85 s) increases in calcium signal, some of which were at the end of seizure and the other were appeared after seizure termination. Approximately 50% of seizures were associated with such signals which were part of wave2s and resembled cortical spreading depression (CSD; Fig. 3A~C, grey box).

CSD was a slowly propagating wave of near-complete depolarization of cells followed by temporary suppression of neuronal activity^20^. Generally, epileptiform calcium signals were characterized by low amplitude and high frequency oscillations accompanied by baseline increase which were like wave1s and calcium build-up of wave2s mentioned before. These CSD-like signals were smooth with rapid rising and slow declining and had no high frequency oscillation. The amplitudes of such waves in different regions were all different from other kinds of epileptiform calcium waves in the same region (Fig. 3A~C). But there was no significant difference of CSD-like signals among different regions.

This strong increase in calcium activity and lasted relatively long time was consistent with a study of CSD that used GCaMP imaging^20^. These CSD-like events did not appear with no epileptic seizure and almost always acted as a signal to seizure termination. But there was no significant correspondence with CSD-like events and behaviors of mice.

## Discussion

In this study, we applied an optical fiber-based calcium signal recording to monitor local calcium activity in hippocampal CA1, CA3, DG and EC in epileptic freely moving mice. The results showed that epileptiform characteristics were found in these four regions which were significantly different from spontaneous signals (before KA administration). Similar to previous studies^21–25^, we recorded one stereotypical pattern of epileptiform calcium activity in all regions, namely, wave1. Wave1 was observed as a small calcium wave with high frequency pulse samples. Wave2 appeared in hippocampus but not in EC and was observed as calcium build-up followed by large calcium wave. Unexpectedly, the amplitude of wave2 in CA3 declined below the baseline and gradually recovered which was different from other regions. There was another special wave that appeared only in CA1 40 min after KA administration, namely, slow flash which was cluster low frequency pulse.

Because the same form of epileptiform characteristic calcium signal was reflected in different brain regions, these calcium signals may not exist in the brain region specificity, but is more related to the different activities of cells caused by calcium ions^26^. More detailed information could be obtained by labeling different types of nerve cells with cell-specific genetic coded calcium-like indicators.

KA could induce robust depolarizations and even cell death^27–29^ and many studies successfully mapped the localization of KA receptors^30^. KA receptors express at different levels in entorhinal cortex^31–33^, cerebellum^34^, amygdale^35^ and basal ganglia^11,36^. And KA receptors also highly expressed in the hippocampus which located both presynaptically and postsynaptically^27^. KA1 subunits are highly expressed in CA3 pyramidal cells, but weakly expressed in CA1^34,37,38^. KA2 subunits are highly expressed both in CA1 and CA3 pyramidal cells^34,37,39,40^. Other KA receptor subunits also contribute to the excitatory action of KA. Therefore, the highly expression of KA1 and KA2 receptor subunits in CA3 region of hippocampus lets it suffer the heaviest damage induced by KA administration^11,41^.

In addition, previous studies have found that following systemic injection, there was a loss of cells in hippocampal CA1, CA3 and CA4, and parvalbumin-positive internerons were also highly sensitive to KA, thus seizures could also affect entorhinal cortex and the subiculum^11^. However there is no direct evidence that calcium waves observed in epileptic seizures are associated with damage or loss of these neurons but the increase of intracellular calcium concentration may lead to excitotoxicity which may result in neurodegeneration. We speculate that the different epileptiform characteristics of calcium activity may caused by the susceptibility of neurons in different brain regions to KA. When waves occurred, mice were accompanied by different levels of seizures. Interestingly, the same kind of waves appearing at different time periods may be accompanied by different behaviors. When different kinds of waves appeared, mice may behave the same way. Therefore, the correspondence between signal and behavior in different regions needs further exploration.

Cortical spreading depression (CSD) is a slow-moving wave that causes almost complete depolarization of brain cells and temporarily inhibits neuronal activity^20^. CSD is characterized by swelling of neurons, marked elevation of extracellular potassium and glutamate concentrations, multiphasic blood flow changes, and decreased tissue oxygen tension^20^. The slow propagation speed of CSD indicates that it is mediated by the diffusion of chemical substances. There is growing evidence that CSD is the basis of migraine aura, and similar waves may appear in apoplexy and tissue damage. The CSD-like signal was recorded in the cortex before^42^. We also recorded this CSD-like activity in hippocampus. Generally, epileptiform calcium signals were characterized by low amplitude and high frequency oscillations accompanied by baseline increase like wave1 mentioned before. These CSD-like signals were smooth with rapid rise and slow decline and had no high frequency oscillation^10,43^. It is notability that we did not observe CSD-like activity in the absence of seizures. Enger *et al*.^20^ found that CSD did not propagate through Ca^2+^ or glutamate, but through K^+^ diffusion in tissue gap. This finding may be used to explain the appearance of this CSD-like event in seizures.

There may be also some limitations that need to be improved in the follow-up study. The surgery required more than 2 h. During this time the OGB-1AM was added and later animals were allowed 1.5 h to recover from surgery. Previous studies have shown that 90% decrement time of isoflurane was 86 min after 6 h of anesthesia^44^. Hence, there may be not much time available for a complete recovery which may make the residual effect of general anesthetics even if its impact was very small. In future experiments, it can be considered to combine GCaMPs and microendoscopic calcium imaging device or other long-term observable equipment. In this way, the animals will have enough time to recover after operation, and the state after administration will be closer to the real physiological phenomenon. Moreover, the GCaMPs can select specific neurons which can further refine the research content.

In clinical diagnosis^45^, patients are often diagnosed with epilepsy in the first or second behavioral seizure^46^. Combined with the results of our study, we inferred that epileptiform lesions have occurred in brain before the occurrence of behavior. We observed epileptiform calcium signals in four brain regions. However, limited to the single channel characteristics of the equipment, it is not possible to detect signals in different brain regions simultaneously in the same brain. Therefore we can only indirectly predict the possible transmission direction of epileptiform signals.

It can be predicted that the sensitivity and high spatial-temporal resolution of fiber optic photometric detection make this method, especially in multiple brain regions of the same animal at the same time calcium signal detection^47,48^, may provide strong support for the pathogenesis of epilepsy and epileptiform signal transmission research^49,50^.

In conclusion, the pathogenesis of epilepsy is very complicated. At present, the pathological features of different brain regions are not yet clear. our research work has recorded the epileptiform calcium signals in different brain regions, and analyzed and classified the stereotypical patterns in these regions, may lead to a better standing of the future direction to drug selection and epilepsy treatment.

## Materials and Methods

### Animals

Adult male C57/BL6J mice aged 8~12weeks, weighing 20~25 g were used in this study. The animals had free access to food and water and were maintained under a 12 h light/12 h dark cycle (lights on at 7:00am). The room temperature was 23 ± 2 °C and the humidity was 50 ± 10%. All experimental procedures were approved by the Animal Care and Use Committee of Tianjin Medical University, in compliance with National Institutes of Health guidelines.

### Drugs

The KA (K0250, Sigma, USA) were formulated before each recording session in 0.9% sodium chloride.

### Seizure assessment

Animals were continuously monitored and scored according to the most severe stage of a modified Racine scale^16^: 0 :no changes in behavior; 1: sudden behavioral arrest and motion less staring; 2: intermittent ear twitches or hiccups, 3: clonus of the head or forelimbs, tail rigidity, 4: generalized clonic seizures with rearing; 5: generalized tonic-clonic seizures with loss of posture and falling. A seizure stage 3 or higher was classified as a convulsive motor seizure (CMS)^13,51^. In this study, the dose of KA (15 mg/kg, subcutaneous) evoked seizure symptoms in most animals. Some reached CMS, but few developed stage 5 seizures.

### Fiber photometry

A custom-built fiber setup was used for the neuronal calcium signal measurements (model “Fiber OptoMeter v2.0”, Suzhou Institute of Biomedical Engineering and Technology; Fig. 1A). The calcium indicator OGB-1AM was excited by a solid-state laser (488 nm). The light intensity of the tip of the fiber was approximately 0.22 mW/mm^2^. The data acquisition and on or off control of the laser light were managed using software on the LabVIEW platform (National Instruments, Austin, TX, USA; Suzhou Institute of Biomedical Engineering and Technology).

### Fluorescent Ca^2+^ indicator staining

The animals were first anesthetized with 2.5% isoflurane in pure O_2_, and then placed on a heating pad with a stereotactic head frame. The animals were continually anesthetized with 0.8%~1.5% isoflurane in pure O_2_ during the surgery process. To prevent drying, animals’ eyes were protected by ophthalmic ointment. After removing hair and skin, a small craniotomy (0.5 × 0.5 mm) was made. The coordinates of the craniotomy were as following (Fig. 1C): the CA1 (from bregma): AP 1.90 mm, ML (relative to midline): 1.00 mm; the CA3: AP 1.90 mm, ML 2.00 mm; and the EC: AP 4.85 mm, ML 3.00 mm; the DG: AP 2.18 mm, ML 1.00 mm. A glass micropipette with a tip diameter of approximately 10μm was filled with OGB-1AM solution and placed directly above the small craniotomy. Approximately 50~100 nl of OGB-1AM solution was injected into the tissue at a depth of 1.25 mm for CA1; 2.10 mm for CA3; 1.75 mm for EC; 2.25 mm for DG (from the cortical surface). Following each injection, the micropipette was kept for an additional 5 min before being slowly withdrawn.

### Calcium recording in freely behaving mice

Approximately 30 min after dye application, an optical fiber was inserted into the same region. The diameter of the optical fiber was about 200μm and the numerical aperture was 0.37. The fiber was glued into a short syringe needle to maintain stability. The optical fiber and skull were fixed together using dental cement. After 20~30 min of solidification, the mice were moved back to their original houses.

Following recovering for approximately 1.5 h, the mice were placed into a transparent round glass cylinder with a diameter of 35 cm in which they could move freely and their performance could be well observed from the outside. A camera was placed just in front of the glass cylinder to record the movements of the mice (Fig. 1B). While the animals were freely behaving, neuronal calcium signals and behaviors were recorded simultaneously. Each mouse was continuously recorded for approximately 2 h. The calcium transients were sampled at 2000Hz with customized acquisition software based on the LabVIEW platform (National Instruments, Austin, TX, USA). The videos were recorded at 30 Hz with a spatial esolution of 1280 × 720 pixels (Logitech C270, Switzerland). All the calcium transients and behavior performances were synchronized offline using event marks.

We used a LED synchronizing calcium signals and behaviors to off-line analyze the correlation between them (Fig. 1B; Suzhou Institute of Biomedical Engineering and Technology). We could light the LED by controlling an output voltage signal in the recording software. In addition to being displayed in the video, LED marks were synchronized in the calcium signal channel.

### Histology and Fluorescence Imaging

After recording, all experimented mice were perfused transcardially with 4% paraformaldehyde (PFA) in phosphate-bufferedsaline (PBS) to document the OGB-1AM staining and confirm the relative position of the optical fiber. Brain samples were post-fixed in 4% PFA overnight. Then, the brain samples were sectioned into 50-um-thick slices. Images were acquired using a inverted fluorescence microscope (BX51, Olympus) and a 4x objective with a numerical aperture of 0.13 (Fig. 4).

### Data analysis and statistics

Calcium transients were acquired at a sampling rate of 2000 Hz after being converting into electrical signals through the Si APD (Avalanche photodiodes). The data were low-pass filtered with a Savitzky-Golay finite-impulse response smoothing filter with 50 side points and a polynomial order of 3. We used relative fluorescence changes, ∆F/F = (f − f_baseline_)/f_baseline_, to represent calcium transients. The f_baseline_ was the baseline level of fluorescence determined during the current recording period of the test. A calcium transient was accepted as a signal when its amplitude was greater than two times the standard deviation of the noise band.

Statistical analysis was conducted in Graphpad prism 7, the experimental results were expressed as Mean ± SEM. We random selected 4 parts signal of each animal as pre-administration signal, the post-administration signal is the epileptiform signal intercepted from each animal sample data, calculating maximum Ca^2+^ signal amplitude (Fig. 2B). For all statistical test, we used Paired t-tests, significance was measured against an alpha of 0.05. The level of *P*  < 0.05 was considered significant.

## References

[1] Singh A, Trevick S (2016). The Epidemiology of Global Epilepsy. Neurol Clin.

[2] Song P (2017). Prevalence of epilepsy in China between 1990 and 2015: A systematic review and meta-analysis. J Glob Health.

[3] Scharfman HE (2007). The neurobiology of epilepsy. Curr Neurol Neurosci Rep.

[4] Delorenzo RJ, Sun DA, Deshpande LS (2005). Cellular mechanisms underlying acquired epilepsy: the calcium hypothesis of the induction and maintainance of epilepsy. Pharmacol Ther.

[5] Ross WN (2012). Understanding calcium waves and sparks in central neurons. Nat Rev Neurosci.

[6] Heuser K (2018). Ca2+ Signals in Astrocytes Facilitate Spread of Epileptiform Activity. Cereb Cortex.

[7] Muir J (2018). *In Vivo* Fiber Photometry Reveals Signature of Future Stress Susceptibility in Nucleus Accumbens. Neuropsychopharmacology.

[8] Selimbeyoglu, A. *et al*. Modulation of prefrontal cortex excitation/inhibition balance rescues social behavior in CNTNAP2-deficient mice. *Sci Transl Med* **9**, 10.1126/scitranslmed.aah6733 (2017). 10.1126/scitranslmed.aah6733 PMC5723386 28768803

[9] Khoshkhoo S, Vogt D, Sohal VS (2017). Dynamic, Cell-Type-Specific Roles for GABAergic Interneurons in a Mouse Model of Optogenetically Inducible Seizures. Neuron.

[10] Berdyyeva TK (2016). Direct Imaging of Hippocampal Epileptiform Calcium Motifs Following Kainic Acid Administration in Freely Behaving Mice. Front Neurosci.

[11] Levesque M, Avoli M (2013). The kainic acid model of temporal lobe epilepsy. Neurosci Biobehav Rev.

[12] Tse K, Puttachary S, Beamer E, Sills GJ, Thippeswamy T (2014). Advantages of repeated low dose against single high dose of kainate in C57BL/6J mouse model of status epilepticus: behavioral and electroencephalographic studies. Plos One.

[13] Tse, K., Puttachary, S., Beamer, E., Sills, G. J. & Thippeswamy, T. Advantages of Repeated Low Dose against Single High Dose of Kainate in C57BL/6J Mouse Model of Status Epilepticus: Behavioral and Electroencephalographic Studies. *Plos One* **9**, 10.1371/journal.pone.0096622 (2014). 10.1371/journal.pone.0096622 PMC4011859 24802808

[14] Kandratavicius L (2014). Animal models of epilepsy: use and limitations. Neuropsychiatr Dis Treat.

[15] Levesque M, Avoli M, Bernard C (2016). Animal models of temporal lobe epilepsy following systemic chemoconvulsant administration. J Neurosci Methods.

[16] Racine RJ (1972). Modification of seizure activity by electrical stimulation. II. Motor seizure. Electroencephalogr Clin Neurophysiol.

[17] Gunaydin LA (2014). Natural neural projection dynamics underlying social behavior. Cell.

[18] Cui G (2014). Deep brain optical measurements of cell type-specific neural activity in behaving mice. Nat Protoc.

[19] Lopez-Madrona VJ, Matias FS, Pereda E, Canals S, Mirasso CR (2017). On the role of the entorhinal cortex in the effective connectivity of the hippocampal formation. Chaos.

[20] Enger R (2015). Dynamics of Ionic Shifts in Cortical Spreading Depression. Cereb Cortex.

[21] Grienberger C, Konnerth A (2012). Imaging calcium in neurons. Neuron.

[22] Daniel AG, Laffont P, Zhao M, Ma H, Schwartz TH (2015). Optical electrocorticogram (OECoG) using wide-field calcium imaging reveals the divergence of neuronal and glial activity during acute rodent seizures. Epilepsy Behav.

[23] Janz P (2017). Synaptic Remodeling of Entorhinal Input Contributes to an Aberrant Hippocampal Network in Temporal Lobe Epilepsy. Cereb Cortex.

[24] Boido D, Jesuthasan N, de Curtis M, Uva L (2014). Network dynamics during the progression of seizure-like events in the hippocampal-parahippocampal regions. Cereb Cortex.

[25] Chauviere L (2012). Changes in interictal spike features precede the onset of temporal lobe epilepsy. Ann Neurol.

[26] Chiang CC, Ladas TP, Gonzalez-Reyes LE, Durand DM (2014). Seizure suppression by high frequency optogenetic stimulation using *in vitro* and *in vivo* animal models of epilepsy. Brain Stimul.

[27] Bloss EB, Hunter RG (2010). Hippocampal kainate receptors. Vitam Horm.

[28] Vincent P, Mulle C (2009). Kainate receptors in epilepsy and excitotoxicity. Neuroscience.

[29] Connell P, Bayat A, Joshi S, Koubeissi MZ (2017). Acute and spontaneous seizure onset zones in the intraperitoneal kainic acid model. Epilepsy Behav.

[30] Ruiz A, Sachidhanandam S, Utvik JK, Coussen F, Mulle C (2005). Distinct subunits in heteromeric kainate receptors mediate ionotropic and metabotropic function at hippocampal mossy fiber synapses. J Neurosci.

[31] Patel S, Meldrum BS, Collins JF (1986). Distribution of [3H]kainic acid and binding sites in the rat brain: *in vivo* and *in vitro* receptor autoradiography. Neurosci Lett.

[32] Xu Z (2016). Entorhinal Principal Neurons Mediate Brain-stimulation Treatments for Epilepsy. EBioMedicine.

[33] Thompson SE, Ayman G, Woodhall GL, Jones RSG (2006). Depression of Glutamate and GABA Release by Presynaptic GABAB Receptors in the Entorhinal Cortex in Normal and Chronically Epileptic Rats. Neurosignals.

[34] Wisden W, Seeburg PH (1993). A complex mosaic of high-affinity kainate receptors in rat brain. J Neurosci.

[35] Rogawski MA (2003). GluR5 kainate receptors, seizures, and the amygdala. Ann N Y Acad Sci.

[36] Jin XT, Smith Y (2011). Localization and functions of kainate receptors in the basal ganglia. Adv Exp Med Biol.

[37] Bahn S, Volk B, Wisden W (1994). Kainate receptor gene expression in the developing rat brain. J Neurosci.

[38] Wondolowski J, Frerking M (2009). Subunit-dependent postsynaptic expression of kainate receptors on hippocampal interneurons in area CA1. J Neurosci.

[39] Castillo PE, Malenka RC, Nicoll RA (1997). Kainate receptors mediate a slow postsynaptic current in hippocampal CA3 neurons. Nature.

[40] Malva JO, Carvalho AP, Carvalho CM (1998). Kainate receptors in hippocampal CA3 subregion: evidence for a role in regulating neurotransmitter release. Neurochem Int.

[41] Levesque M (2009). Synchronized gamma oscillations (30-50 Hz) in the amygdalo-hippocampal network in relation with seizure propagation and severity. Neurobiol Dis.

[42] Zhang Q (2017). Locomotion-Related Population Cortical Ca(2+) Transients in Freely Behaving Mice. Front Neural Circuits.

[43] Jennings JH, Stuber GD (2014). Tools for resolving functional activity and connectivity within intact neural circuits. Curr Biol.

[44] Bailey JM (1997). Context-sensitive half-times and other decrement times of inhaled anesthetics. Anesth Analg.

[45] Babb TL (1996). Glutamate AMPA receptors in the fascia dentata of human and kainate rat hippocampal epilepsy. Epilepsy Res.

[46] Thom M (2014). Review: Hippocampal sclerosis in epilepsy: a neuropathology review. Neuropathol Appl Neurobiol.

[47] Guo Q (2015). Multi-channel fiber photometry for population neuronal activity recording. Biomed Opt Express.

[48] Allen WE (2017). Global Representations of Goal-Directed Behavior in Distinct Cell Types of Mouse Neocortex. Neuron.

[49] Lu Y (2016). Optogenetic dissection of ictal propagation in the hippocampal-entorhinal cortex structures. Nat Commun.

[50] Lutcke H (2010). Optical recording of neuronal activity with a genetically-encoded calcium indicator in anesthetized and freely moving mice. Front Neural Circuits.

[51] Puttachary S (2015). Immediate Epileptogenesis after Kainate-Induced Status Epilepticus in C57BL/6J Mice: Evidence from Long Term Continuous Video-EEG Telemetry. Plos One.

